# A collaborative multi-university virtual model for global health leadership education integrating educational technology: a mixed methods evaluation

**DOI:** 10.3389/fpubh.2026.1706228

**Published:** 2026-02-24

**Authors:** Shubha Kumar, Catherine Zhou, Mellissa Withers

**Affiliations:** 1 Department of Population and Public Health Sciences, University of Southern California, Los Angeles, CA, United States; 2 Office of the Dean of Engineering, The Hong Kong University of Science and Technology, Hong Kong, Hong Kong SAR, China

**Keywords:** AI-use, education, educational technology, evaluation, global health, leadership

## Abstract

Challenges and constraints in global health practice such as inadequate international cooperation, cultural sensitivity, and interdisciplinary collaboration inform key gaps and opportunities to strengthen global health education, including technical skills and competencies needed for the next generation of global health leaders. We describe an innovative model of global health education leveraging advances in educational technology, co-developed and implemented by universities within the Association Pacific Rim Universities. The model was developed to address challenges and opportunities to strengthen global health education and has been implemented in two global health courses—one on leadership and one on ethics—for almost 10 years. The model was innovative in providing a virtual global learning environment in collaboration with multiple universities in diverse contexts, a novel approach back in 2015 and one that to this day is not widely available across universities. An evaluation of the model was conducted in 2025, and findings are reported. To evaluate the global health education model, we applied a mixed methods approach including surveys and discussion groups with students and faculty participating in the global health leadership course in the past 2 years. Approximately 83% of students (148/179) responded to the student survey and more than 80% of respondents reported high levels of mastery of course learning objectives and competencies, while 97% of students reported satisfaction with the course, with an emphasis on the model’s benefits for developing and practicing global collaboration and communication. Faculty reported high satisfaction with the course’s inclusion and modelling of a decolonized approach to global health education with the diversity of participating learners, guest speakers, and case studies and readings in the course. Developing and implementing effective global health education is critical for improving education and practice. Our intention in sharing this case study is to offer readers the opportunity to learn about and adapt this model in their own curricula and settings as desired as we collectively strive for improved global health education. The more such models are implemented across various topics and settings, the more we can achieve increased scalability and success in key components of global health training and practice.

## 1 Introduction

Global health is an area of study, research, and practice. Reflecting on global health practice and research is critical to inform approaches to global health education. Global health aims at improving health and achieving equity in health for all people worldwide. It emphasizes transnational health issues, determinants, and solutions. It involves many disciplines within and beyond the health sciences and promotes interdisciplinary collaboration (1). This definition points out the key ingredients of global health: ‘global’, ‘transnational’ and ‘interdisciplinary’. ‘Global’ aims at health for all people, irrespective of their nationality or location (2). ‘Transnational’ indicates discussion, communication, and collaboration across countries.

Major challenges and constraints continue to persist in global health practice, particularly in low- and middle-income countries (LMICs). Challenges remain in developing and obtaining the internal capacity, financial and human resources needed to support people in real time and especially in LMICs, as well as the transfer of such resources between countries. Additional constraints include lack of information, low levels of literacy, low quality of care, and poor local infrastructure in many LMIC settings. Furthermore, research and development (R&D) is usually carried out in the for-profit sector, where organizations often express concerns regarding the sufficiency of return from products oriented towards LMICs. This causes insufficient attention to the local health conditions of LMICs and makes it more challenging to apply R&D outcomes in those countries (3). To address such issues, global health education must focus on technical skills needed to address such challenges. It should raise students’ awareness of global outlook and encourage collaborative transnational research and practicums (4). More importantly, it should provide a context-based learning environment that prepares students with the competencies needed in resource-constrained settings (5).

‘Interdisciplinary’ collaboration is essential in global health. Workforces across different fields can and should work together to develop solutions to global health challenges, such as health financing, road safety, violence prevention, health workforce, and air pollution (6). This collective effort requires global health professionals to be equipped with such interpersonal competencies as communication, teamwork, and leadership. Furthermore, evidence-based leadership development has become increasingly essential in healthcare settings, as leadership decisions grounded in structured evidence rather than intuition are linked to improved system performance and stakeholder outcomes (7). Global health education should provide students with authentic learning opportunities to practice these skills in diverse teams (8).

Considering the nature and challenges of the current global health landscape, the importance of competency-based training across institutions is clear (9). Competency-based education prepares students to demonstrate how they would contribute effectively to the workforce upon graduation (10). Global health programs supported by a competency-based model help institutions prepare the next generation of global health leaders and ensure adequate training no matter where they work (9). Take the Global Health Competency Model (10) for example, experiential learning approaches such as case studies and simulations are able to provide real-life examples of global health challenges and solutions and help students with competency building through practicums (9). While a number of key global health competencies have been identified, opportunities to practice some of these competencies such as demonstrating diplomacy and building trust with partners or approaching global partnerships with cultural humility and respect (11) can be challenging to practice without opportunities for students to interact with international colleagues. To link students in different settings, appropriate use of available technology should be considered to create interactive and authentic learning environments, which will facilitate cross-institution experiential learning in today’s technology-dominated world (9, 12).

Furthermore, examination of the ways in which global health may reflect colonialist ideals has been the subject of a wide body of literature in the past decade (13–17). Global health, and its previous iterations of “tropical medicine” and “international health,” stemmed from colonialism and its early emphasis on western biomedical models to ‘civilize’ populations living in low-income countries. The biomedical model of health and disease was viewed as superior and necessary to suppress the perceived threat of infectious disease transmission to high-income countries (HICs). However, these models privileged western viewpoints and disregarded the value and potential contributions of non-western forms of healing (14, 18–22). The power imbalances created through colonialism persist in global health education and training. They are represented in numerous ways such as the proliferation and perceived value of information coming from the Global North versus Global South. Curriculum is often in English and relates to scenarios that are relevant in high-income contexts without consideration for the local resources and culture, and often excludes the knowledge of indigenous and non-western groups (13, 15, 23–27). Another example is unequal power dynamics in global health short trips and exchange programs, where partners from the Global North often receive disproportionate benefits while the Global South partners bear most of the burden and costs, including increased use of physical resources, stretched responsibility to provide oversight to new students, and a lack of reciprocal opportunities or bi-directional exchange (13). Students may fail to learn from or integrate into the host context (13, 14, 28, 29).

Many scholars have highlighted the need to decolonize global health education (13, 15, 17, 25, 30–32). Part of training in global health should include critical thinking skills to question such norms and biases, and to encourage the goals of a more equitable world (15). A new paradigm is needed to go beyond the traditional global health curricula to teach broader concepts and skills of ethical and social responsibility, global citizenship, and leadership. Meaningful cross-cultural connections with students in other countries can be transformative (33). Innovations in technology and interest in internationalization of curricula have dramatically transformed the educational landscape. New ways to facilitate experiential learning and cross-cultural connections exist through virtual platforms, which can help to address some of the inequities in access to traditional international exchange programs (34–37).

We present a case study of an innovative model of global health education which responds to many of the key challenges and opportunities described above, along with an evaluation of this approach. The model was innovative in that it provided a virtual global learning environment in collaboration with multiple universities in diverse contexts, a novel approach back in 2015 and one that to this day is not widely available across higher education institutions.

## 2 Pedagogical design and implementation

The global health educational model was co-developed and implemented by universities within the Global Health Program of the Association of Pacific Rim Universities (APRU), a non-profit network of 60 + leading research universities in the Asia-Pacific. A collaborative international inter-university model was pursued to provide students with opportunities to put into practice the application of key global health leadership competencies, for example communication with diverse partners and cultural humility. In 2015, faculty from a handful of APRU universities co-conceptualized the model, leveraging advances in online education and technology to create a global learning environment. Faculty from one university (the University of Southern California) took on primary responsibilities for both the design and implementation of joint educational resources (i.e., a shared learning management system, shared weekly schedule, instructions for group assignments, etc.) and the coordination amongst participating universities.

A decade ago, synchronous distance education learning was for the most part still in its infancy. Platforms such as Zoom were not widely used. If even available, most online courses were asynchronous and confined to single institutions. Coordinating curricula, technology platforms, academic standards, and time zones across countries required both technical sophistication and institutional support for new ideas. Further, developing such models was challenging because they required significant cooperation across institutions in different countries; differences in accreditation systems, language, pedagogy, and digital infrastructure made such collaboration complex and rare. Successfully delivering shared learning experiences to geographically dispersed students including from many different disciplines demonstrated an early vision of global education, showing that knowledge could be co-created and accessed beyond borders well before international virtual campuses and large-scale global online programs became mainstream.

While the global health education model presented here was developed and began to be implemented well before the Collaborative Online International Learning (COIL) Virtual Exchange (VE) COIL+OSCQR standards were released, the underlying pedagogical approach of the model is well aligned with these standards (38). COIL VE is a unique way of incorporating an international experience into a curriculum as learners engage with peers from another country, culture, and context (39). The COIL+OSCQR standards reflect research-based pedagogical approaches and best practices focused on virtual international exchange and the design of intercultural online learning experiences (38). The standards suggest: (1) Learners engage in scaffolded opportunities for communication among intercultural virtual team members (intercultural communication); (2) Learners engage in activities that help them recognize their partner’s cultural values, beliefs, and biases, as well as their own (cultural awareness); (3) Learners construct a culturally collaborative and respectful dialogue in an international virtual team, both verbally and non-verbally (collaboration); and (4) Learners in cross-cultural groups build skills to organize and distribute the work in asynchronous and synchronous technology platforms (teamwork skills). Reflecting upon course practices and assignments, it became clear that the model we developed met all key criteria of the standards.

## 3 Learning environment

This model has been applied in two different semester-long graduate courses, one focused on global health leadership and another on global health ethics. In each course (which have been offered on an annual basis since 2015 and 2016 respectively), 4–7 universities from across the Asia-Pacific come together online over the course of a semester for joint learning. In the leadership course for example, a combination of the following universities have participated over the years: Osaka University, Fudan University, Peking University, University of the Philippines, Chulalongkorn University, The Chinese University of Hong Kong, Nanyang Technological University, National Taiwan University, Tecnológico de Monterrey, University of California Irvine, and University of Southern California. Typically, 60–100 students across all of the participating universities enroll in each course. Each course consists of weekly virtual guest lectures from global health leaders and practitioners in the field, including those working at the World Health Organization (WHO) and other United Nations (UN) agencies, country governments, non-government organizations (NGOs), and more. The guest lectures offer students the unique opportunity to hear from a diverse group of experts who are often not in reach of a single faculty or university alone. The guest lectures are followed by Q&A, case study presentations and discussions, and group assignments where students from each of the universities are mixed together for a collaborative and diverse learning experience. Both courses employ a competency-based approach and include specific learning objectives as relevant to global health leadership or ethics, respectively (40). Creation of a global learning environment through the online multi-institutional model is particularly helpful in providing students with opportunities to practice specific competencies relevant to global health leadership and ethics (for example, intercultural communication, cultural humility, and appreciation for diverse perspectives) that they may not otherwise have in a traditional, single university classroom.

The learning environment and key practices employed in the model were intentionally designed to support a decolonized approach to global health education. In addition to the deliberate inclusion of geographically and culturally-diverse universities, faculty, and guest speakers, numerous case studies and readings were assigned throughout the courses from authors in the global North and global South, and on issues such as power differentials in the North and South. In addition, faculty from each university/region equally facilitated small group discussions in breakout rooms which specifically included a mix of students from each university/region to enable rich discussion from diverse perspectives. Furthermore, during the small group discussions, the facilitators specifically addressed themes of decolonization. In addition, during the group presentations, groups presented on the ways that the case studies highlighted these themes. The leadership course also dedicated an entire week specifically to the topic “decolonizing global health.”

While there were a few challenges implementing the model, all were able to be overcome. First, to manage time zone differences, it was agreed that the course would be offered at a time suitable for students across countries to attend synchronous class (this meant early evening time for students in the US and Mexico and morning time for students in Asia). Second, to manage differences in academic calendars, the course was limited to 10 weeks during the semester where all universities had overlap. (For any universities that had longer semesters, they implemented their own curriculum during those additional weeks.) Third, to manage matters of tuition, it was agreed that there would be no shared tuition or complicated sharing arrangements, rather that each university would collect tuition from its own students as it typically does.

## 4 Results and assessment

**Figure 1:**
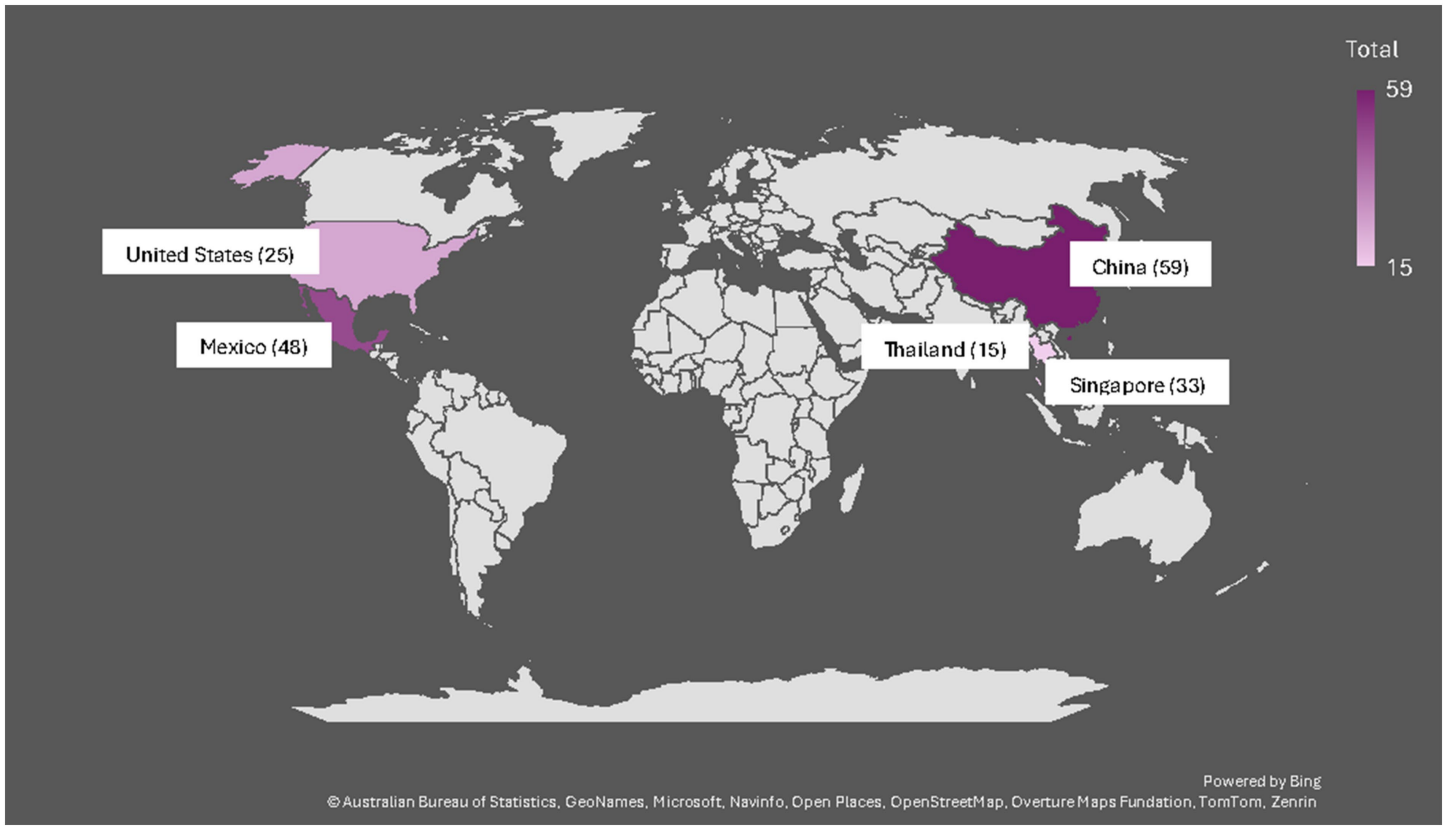
Number of students enrolled from each country across 2023 and 2024.

An evaluation of the model was conducted in 2025. Due to resource constraints, the (formal) evaluation focused on one of the two courses—the global health leadership course. To evaluate the global health education model, we applied a mixed methods approach including surveys and discussion groups with students and faculty participating in the global health leadership course in the past 2 years (for a visual overview of student enrollment from each country, see Figure 1). We developed two feedback surveys—one for students and one for faculty—and administered the surveys online using Qualtrics, at the end of the course in 2023 and again in 2024. Faculty from the coordinating university collaborated to develop survey items focused on assessing student and faculty experiences and perceptions of the course model. The surveys consisted of both open-ended and closed-ended questions using a 5-point Likert scale approach. The same survey instruments were used in both years, with the exception of additional questions integrated in the 2024 version of the student survey given the rapid proliferation of generative AI (GenAI) to additionally understand how students were using AI in the course. The questions related to GenAI were developed based on common knowledge of uses of GenAI by students in higher education and grouped together as an additional section at the end of the 2024 survey, after the consistent set of questions from the 2023 survey. Surveys were sent to all students for their anonymous feedback, as well as to faculty, respectively. In addition to the surveys, faculty held structured discussions (using a common set of questions) with students in breakout rooms at the end of the course each year to gain additional perspective and insights from the students regarding their experiences in the course and any suggestions for improvement. Faculty took notes during these sessions and the authors reviewed all notes for any themes that emerged across the discussions.

### 4.1 Evaluation findings from students

Results from the student surveys reflect students’ feedback with respect to mastery of learning objectives, competencies and qualities developed in the course, and perceived effectiveness of instructional approaches. Response rates were 87.4% (83/95) in 2023 and 77.4% (65/84) in 2024.

#### 4.1.1 Mastery of learning objectives

**Figure 2:**
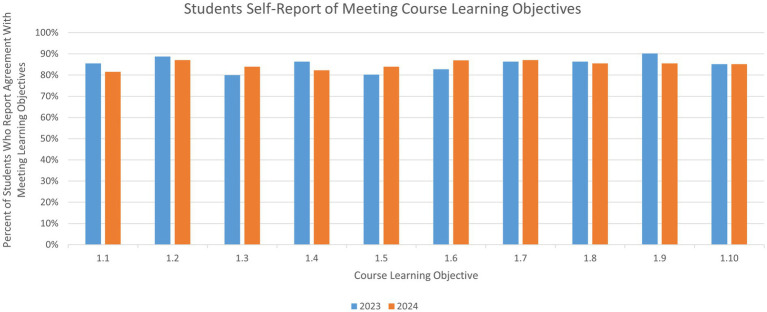
Students self-report of meeting course learning objectives.

Across the surveys of all participants in both years, more than 80% of the 148 respondents reported they agree (either strongly agree or somewhat agree) that they have developed the abilities required for global health leaders (the key learning objectives of the course), including but not limited to analyzing key issues in global health agency management, describing various components and functions of a health system, and coordinating diverse stakeholders (1.1–1.5 in Table 1). More than 82% of the 148 respondents reported being equipped with the ability of identifying critical leadership traits (1.6 in Table 1). Over 85% reported having developed the abilities essential for global health professionals, such as identifying key challenges in resource-limited settings and discussing equity and inclusion in global health (1.7–1.10 in Table 1) (for a visual overview of Table 1 results, see Figure 2).

**Table 1:** Responses to question “by the end of the course, I have developed the abilities to…”

Course learning objectives	2023 (no. of respondents: 83)	2024 (no. of respondents: 65)
	Agree	Agree
1.1 Analyze key trends and issues in management of global health agencies and organizations	85.5%	81.5%
1.2 Explain the importance of cooperation in global health and explain the ways that diverse players in government, the private sector, non-governmental organizations and multi-lateral organizations can work together or individually to impact the health of a community	88.8%	87.1%
1.3 Describe the current and historical role of the World Health Organization in global health governance and the various strategies that have been suggested for how to improve it	80.0%	83.9%
1.4 Describe the sustainable development goals and how they relate to global health	86.4%	82.3%
1.5 Describe the various components of a health system and their overall functions	80.2%	83.9%
1.6 Identify critical traits that contribute to successful leadership and past and current leaders that exhibit these qualities	82.7%	86.9%
1.7 Identify key challenges in developing and implementing health programs in resource-constraints settings	86.4%	87.1%
1.8 Discuss the critical shortage of health care workers in developing country settings, including the cause and impact	86.4%	85.5%
1.9 Discuss the importance of equity and inclusion in global health	90.2%	85.5%
1.10 Discuss examples of key successful global health case studies	85.2%	85.2%

Analysis of qualitative responses provides further insights into students’ learning in these areas. For example, related to equity and inclusion, one student remarked, “*This course … reminded me that progress is not only about cutting-edge and fancy technology or culture; it is also about the everyday well-being of large, often overlooked populations. True change requires social action and clear leadership that can rally people, raise awareness and turn attention into collective effort*.*”*

Another student commented on inclusion in resource-limited settings, “*The lecture that impressed me the most was on cultural adaptation and health system reform in global health. The lecturer shared vivid examples of how integrating local cultures and practices can enhance the effectiveness of health interventions in resource-poor areas. This not only made me re-examine the way global health is implemented, but also made me realize that cultural sensitivity and inclusiveness are key to developing sustainable solutions in the context of globalization*.”

#### 4.1.2 Competency development

In addition to reflection on whether students met the learning objectives of the course, students were also asked to reflect on their development of course competencies. The development of cultural and emotional competencies is implicit as the basis for the success for global health programs (4). More than 88% of the 146 respondents across both courses in 2 years reported that they somewhat agree or strongly agree that they have developed cultural sensitivity and appreciation of diversity (2.1–2.4 in Table 2).

**Table 2:** Responses to question “this course helped me develop these qualities.”

Core competencies	2023 (no. of students: 83)	2024 (no. of students: 63)
	Agree	Agree
2.1 Appreciation of diversity, equity, and inclusion	88.9%	90.3%
2.2. Global collaboration	88.9%	90.3%
2.3 Cultural sensitivity/ humility	88.0%	88.9%
2.4 Appreciation of others’ viewpoints and opinions	91.4%	88.7%
2.5 Critical thinking	82.7&	88.7%
2.6 Problem solving	80.2%	82.3%
2.7 Ability to work in a team	87.7%	90.5%
2.8 Verbal communication	86.6%	87.3%
2.9 Written communication	72.8%	85.5%
2.10 Public speaking	79.3%	87.3%
2.11 Presentation skills	82.7%	87.1%
2.12 Active listening	87.7%	88.7%

Qualitative findings strongly suggested that students felt that the exchange of ideas and learning about different viewpoints across cultures was incredibly useful for their future careers. For example, one student mentioned, “*The collaboration with students from around the world was incredibly valuable, as their diverse perspectives enriched our discussions on important global issues. This course challenged me to think beyond my own Western views, and I feel my global perspective has truly expanded and evolved as a result*.”

**Figure 3:**
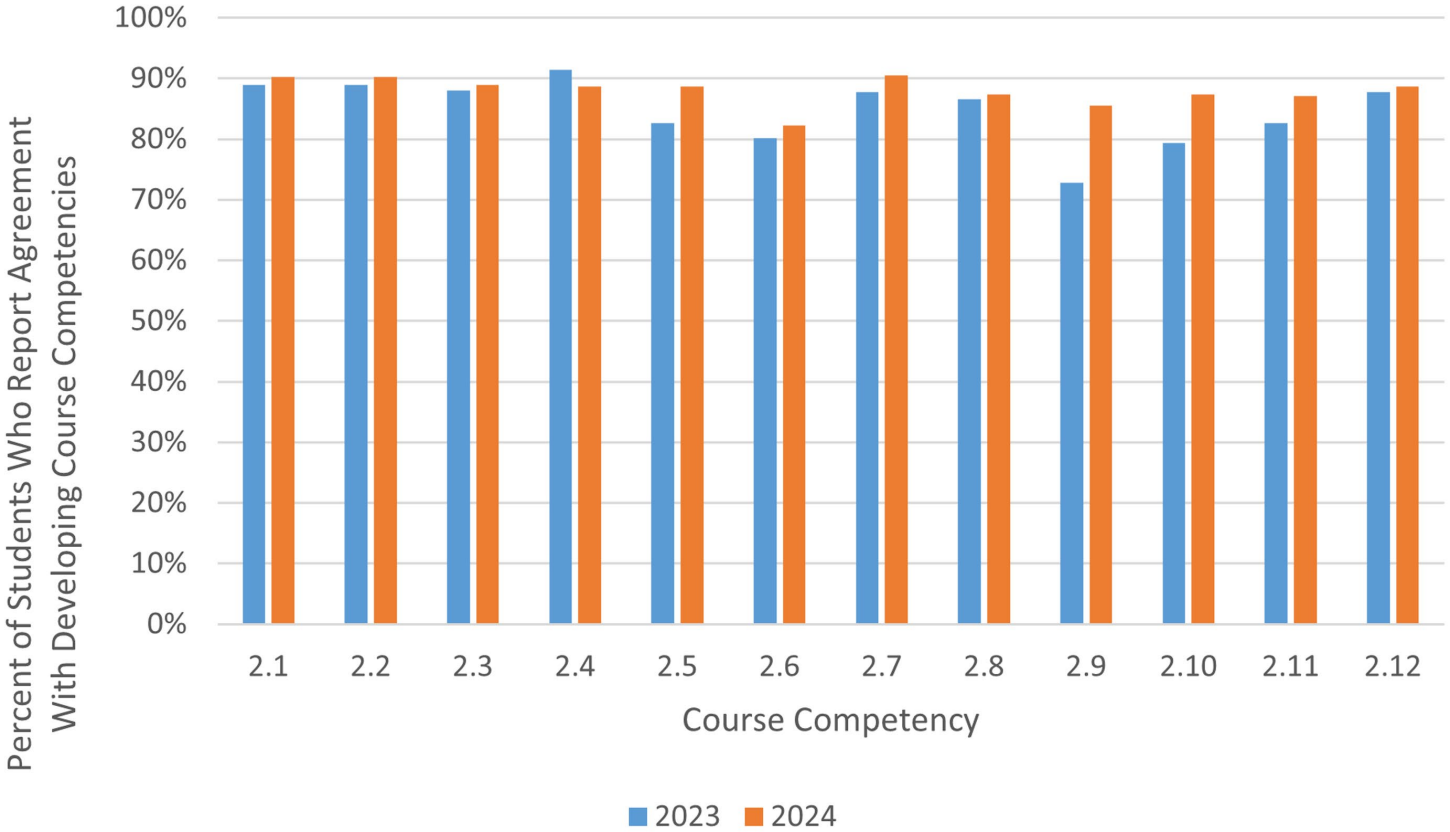
Students self-report of developing course competencies.

In addition, more than 80% reported they believe that they have developed the interpersonal competencies that global health professionals need to possess, such as critical thinking, problem solving, teamwork, active listening, and communication (2.4–2.12 in Table 2) (for a visual overview of Table 2 results, see Figure 3).

Students felt that they were able to develop new skills from the group presentations and small group discussions, such as communication and improved English language fluency. For example, one student explained, “*The main lesson I am taking away from this class is the importance of communication. No matter the time or language barrier, communicating with others is such a crucial and significant skill to possess, and learning how to work in a group despite any differences in backgrounds or cultures is especially important in a global health context. While I feel that all the groups I was in this semester really excelled in communication, I can always improve on my own skills and I will apply the knowledge I gained from working with all the wonderful students and teaching assistants this semester to my future goals through cultivating my active listening, responsibility, and teamwork skills*.”

As these results on mastering course competencies are self-reported and cross-sectional, they do not necessarily reflect actual mastery or change over time, nor faculty assessment of students’ performance, rather they indicate student’s perceptions of their own learning by the end of the course and within this unique learning environment.

#### 4.1.3 Perceived effectiveness of instructional approaches

**Figure 4:**
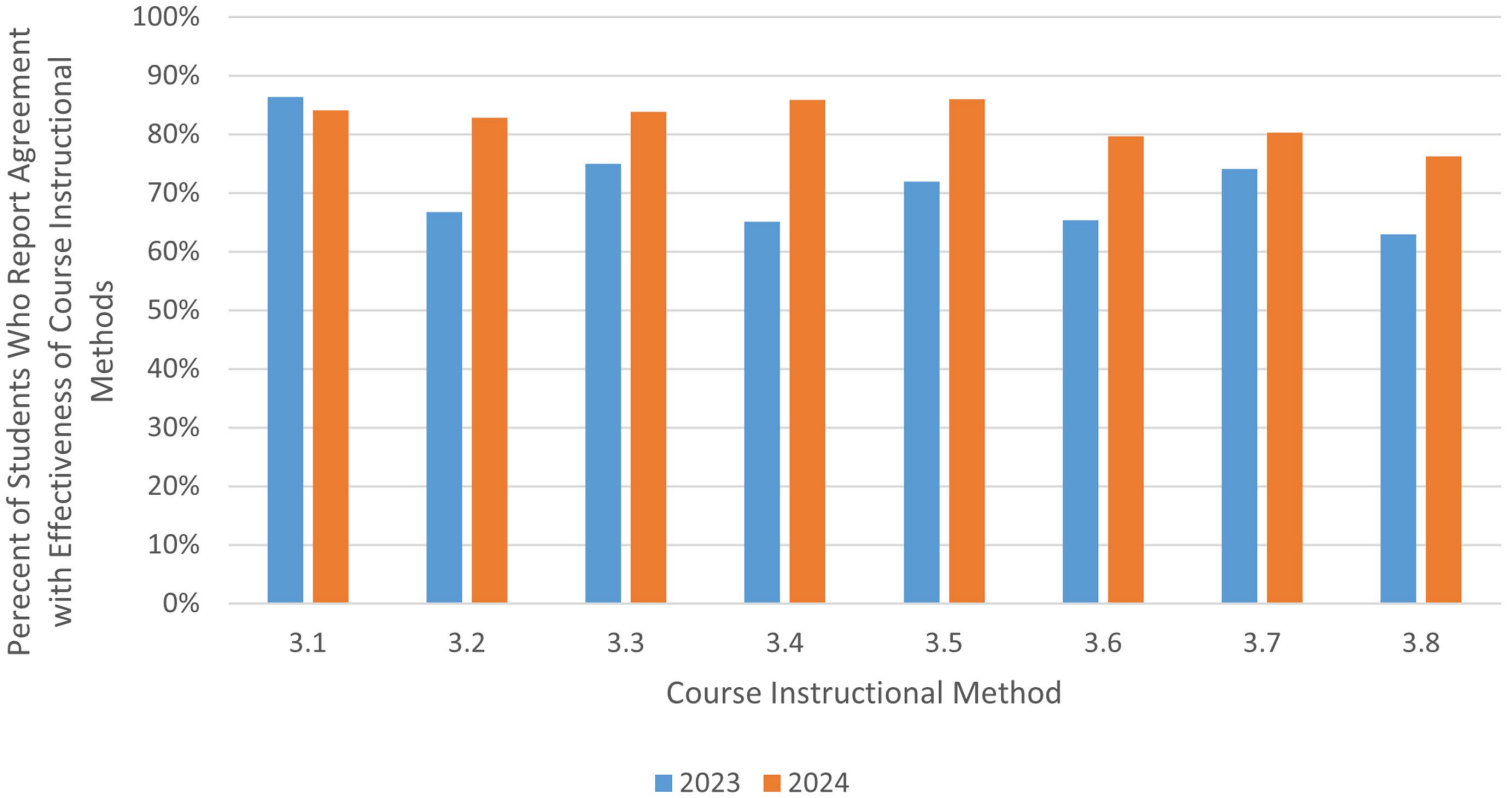
Student feedback on course instructional methods.

Case studies and simulations are key learning and assessment approaches used in the course, coupled with presentations and group discussions. More than 80% of the 63 respondents in the 2024 class reported they somewhat agree or strongly agree that these activities effectively helped them learn and practice both technical skills and interpersonal competencies (3.1–3.5 in Table 3) (for a visual overview of Table 3 results, see Figure 4).

**Table 3:** Responses to “how effective do you think [the teaching methods] were?”

Course instructional methods	2023 (no. of responses: 83)	2024 (no. of responses: 63)
	Agree	Agree
3.1 Analysis of case studies on global health leadership from textbook	86.4%	84.1%
3.2 HBS case studies	66.7%	82.9%
3.3 Online simulation (patient zero)	75.0%	83.8%
3.4 Small group class discussions	65.1%	85.9%
3.5 APRU group presentations	71.9%	86.0%
3.6 Discussion boards to help facilitate group discussion and individual reflection	65.4%	79.7%
3.7 Online training on cultural competency	74.1%	80.3%
3.8 Individual exercises and homework assignments to practice concepts and skills	63.0%	76.2%

For example, one student reflected, “*The small group discussions were very helpful; as it makes it an exercise for developing personal and social skills at the same time technical skills as we discussed more thoroughly the cases*.”

Another student remarked, “*The main lesson I will be taking away is what it means to work in a group with people from different backgrounds, communication styles, and experiences. I tend to approach group projects with a single-minded approach, but I needed to learn how to consider the working styles of everyone in my group. This is especially important in the professional world, where I may be collaborating with people from all over the world. I hope I take this newfound perspective in whatever teamwork I do. I’m not always right, and I need to listen and collaborate with an open ear*.”

In addition to the above course methods, the opportunity to learn from renowned and diverse guest speakers was another major highlight of the course, according to students.

For example, one student explained “*I believe our course has provided me with a unique opportunity to gain insights into perspectives of leaders and professors from various cultures within the global health arena, offering me a window to explore the world. This exposure has not only broadened my understanding of diverse health issues but also underscored the importance of cross-cultural collaboration in addressing global health challenges. [… One of [the] speaker[s]] said that we should first fully understand the [needs of] special groups. I think this will give me some inspiration for my later study on the issue of health equity, because equality is not equal to fairness*.”

Other students mentioned the benefits of interacting with leaders who had on-the-ground experience in addressing global health challenges in practice. For example, one student commented “One of the most valuable aspects of this course was gaining diverse perspectives from various speakers on the global health issues facing our world today. Through their insights and expertise, I developed a deeper understanding of the complexities and challenges in addressing public health concerns across different regions, as well as the innovation solutions being implemented to tackle these issues.”

#### 4.1.4 Use of GenAI

**Figure 5:**
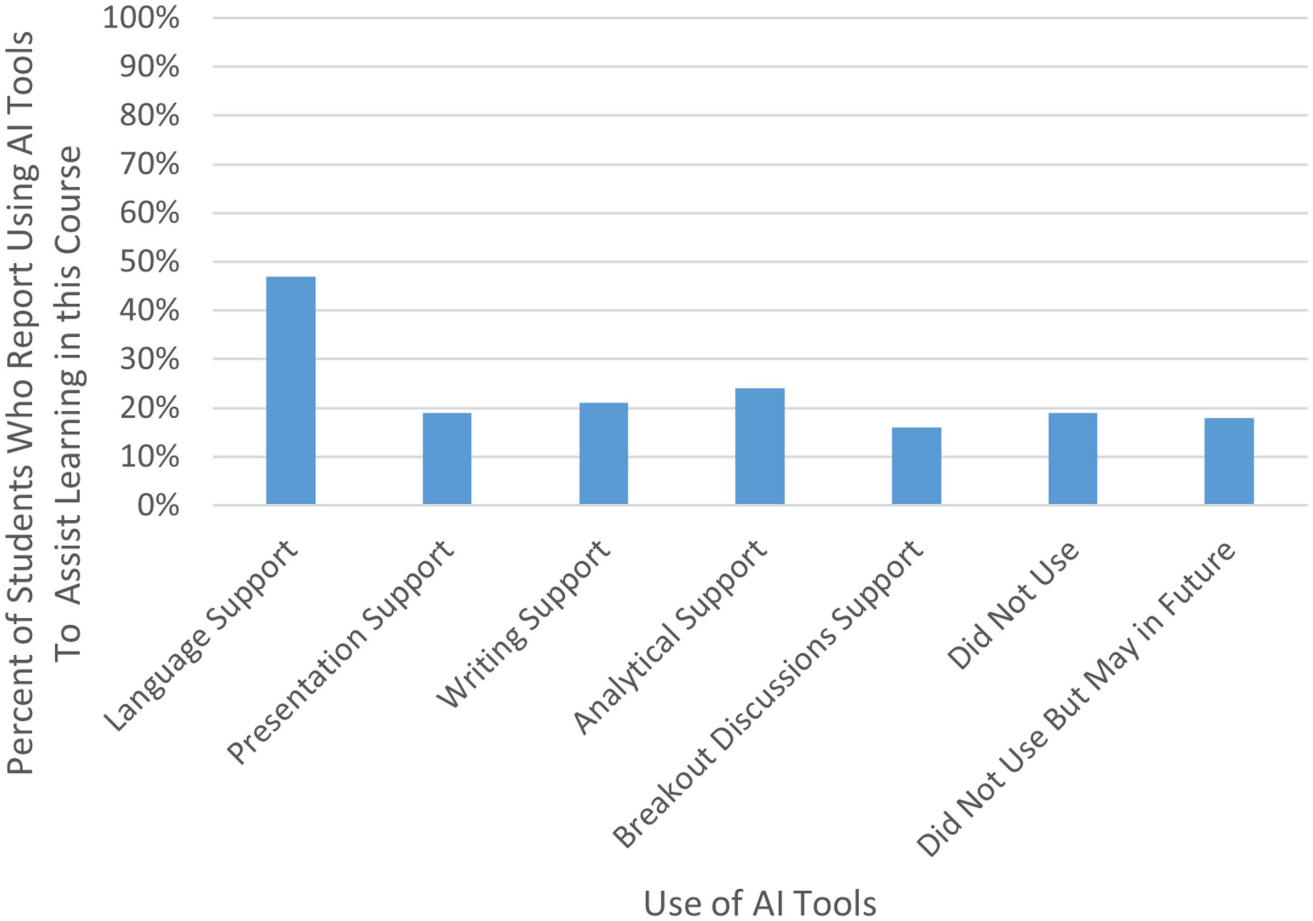
Use of GenAI tools by students in 2024 cohort.

Given the increasing use of GenAI technologies such as ChatGPT among students coupled with the unique structural model of this course, we also sought to understand how this global cohort of students may be using GenAI to assist with their learning in this course, in the 2024 survey (41). Less than half of the 63 students reported using GenAI to assist with their learning in this course, and of those that did, the most common use of GenAI technologies was to support with language translation and/or transcription (see Table 4) (for a visual overview of Table 4 results, see Figure 5).

**Table 4:** Responses to “how did you use AI tools to assist with your learning in this course?”

Use of AI tools	2024 (no. of responses: 62)
	Percent of students who reported use
4.1 Assistance with language translation and/or transcription	47%
4.2 Assistance with creating presentations	19%
4.3 Assistance with written assignments	21%
4.4 Assistance with analysis for assignments	24%
4.5 Assistance with responding to breakout room questions	16%
4.6 I did not use any AI tools to assist with my learning in this course	19%
4.7 I did not use any AI tools to assist my learning in this course but think I may in the future	18%

#### 4.1.5 Overall findings from students

Overall, 96 and 97% of total 148 respondents (in 2023 and 2024 respectively) reported they would recommend the course to other students. Taken together, these results along with the high response rate to the surveys (87 and 77%) and the high percentage of agreement (over 80%) across responses indicate student appreciation of the course model. With respect to suggestions for improving this course, key themes which emerged included: mixed feedback on discussion boards, incorporating additional topics, and offering opportunities for excursions or field trips as part of the course.

In addition to results from the student surveys, findings from the structured discussion sessions with students largely reinforced survey findings and shed insights regarding specific guest lectures students most appreciated, with the lecture on decolonizing global health in particular.

### 4.2 Evaluation findings from faculty

Results of the faculty surveys reflect faculty feedback regarding the range of disciplines represented by students enrolled in the course, students’ key takeaways from the course, the benefits afforded to students by this unique course model, and faculty perceptions regarding the diversity of guest speakers. Response rates were 16.7% (2/12) in 2023 and 57.1% (8/14) in 2024.

#### 4.2.1 Interdisciplinary collaboration

A wide range of academic disciplines were represented in the course in both years. Results indicated there were students participating from degree programs such as public health, medicine, dentistry, psychology, health administration, engineering, business, and food safety. These results directly relate to another result from the faculty surveys: learning how to collaborate with learners from diverse perspectives during group discussions and projects as both one of the students’ key takeaways from the course and one of the main benefits offered by the course model, which is essential to such interdisciplinary fields as global health.

As one student had remarked, “*This experience enables me to work with people who have different cultural backgrounds to exchange ideas and allow us to discuss real-world global health challenges from diverse cultural perspectives. This course also provides me with the first experience of collaborating with students with the same goals and global health experience. The collaboration enhanced our real-world experience and expanded my horizons on how different regions address the same global health challenges*.”

#### 4.2.2 Appreciation for diverse perspectives

Additional results from the faculty survey as related to students’ key takeaways from the course largely mirrored the results from the students’ surveys, with an emphasis on the appreciation for others’ viewpoints, cultural sensitivity/humility, and critical thinking skills (2.1–2.5 in Table 2).

In terms of modelling geographic and cultural diversity and a decolonized approach to global health education, faculty survey results indicate strong satisfaction with respect to the diversity represented by guest speakers, with an average of 8.75 out of a 10-point scale.

#### 4.2.3 Recommendations for future implementation

With respect to the open-ended question regarding any suggestions for improving this course, key themes which emerged included: integrating additional topics in the course, as well as additional guest speakers as feasible.

## 5 Discussion and conclusion

This case study presents an innovative model of global health education, including an illustration of how it addresses key challenges and opportunities in global health training as evidenced in the literature, along with an evaluation of its effectiveness from the perspectives of those involved. Evaluation results suggest the model is promising in addressing the needs for transnational and interdisciplinary education in global health and competency-based education in global health, including an emphasis on cultural and interpersonal competencies in addition to technical skills. Furthermore, the course not only facilitates a rich understanding and discussion of decolonizing global health, but the model itself puts into practice a decolonized approach to global health education with the diversity of participating learners, guest speakers, and assigned case studies and readings in the course.

Collectively, the components of the global health education model presented in this case study are suggestive of its relevance and effectiveness in preparing the next generation of global health leaders. These findings align with the wider literature demonstrating that leadership capacity is strengthened when learners are exposed to structured, evidence-based decision-making models embedded within training environments (7), such as in the course case studies and other group assignments.

Key practical implications and lessons learned from the model’s implementation over nearly a ten-year period include (1) the need for there to be at least one designated university responsible for coordination across participating universities, (2) scheduling challenges can be overcome with careful consideration of all participants’ academic calendars, time zones and the willingness of guest speakers, and (3) that advances in educational technology continue to make such collaborations easier and more interactive. Furthermore, while there may be differences in pedagogical approaches used in different cultures, this did not pose any significant challenges in the pedagogical approach implemented in this model. And while language barriers may have presented challenges for some students who were not as comfortable with spoken English, the emergence of AI tools for language translation and transcription can now support such students in communicating more easily.

There are some limitations of the case study, most notably that the evaluation results are based on self-report and that halo bias may be present. In addition, the model was implemented within a specific consortium of Asia-Pacific universities, which may not reflect the structural, technological, or cultural realities of institutions in other regions. The study does not compare outcomes by nationality, socioeconomic background, academic discipline, or prior online learning experience, which may mask inequities in engagement and benefit. Other than course assignments, leadership development was assessed only through self-reported perceptions without using validated leadership assessment tools, control groups, or longitudinal follow-up to measure actual competency acquisition and real-world application. In addition, students with stronger internet connectivity, device access, and prior experience with online platforms and AI tools may have had an advantage, yet no baseline comparison or control was included. However, despite these study limitations, overall findings suggest this model of global health education can be effective in multiple ways and merits further exploration, implementation, and evaluation.

In addition, this case study contributes to the evidence base on use of GenAI in higher education. Within this unique education model, students reported most using GenAI tools for support with language translation/transcription. However, overall results on the use of AI tools from this study reflect relatively low use and/or hesitancy in using GenAI tools in their learning, aligning with existing studies which have highlighted similar and/or mixed results (42). As this study captures only initial adoption of GenAI tools during a single academic year (2024), trends in AI usage cannot be interpreted longitudinally or linked to learning outcomes, though if and how these results change over time will be worth tracking.

In considering future directions and implementation of this education model, it is also worth delving into how faculty can leverage GenAI tools to enhance global health leadership education within this model and more broadly. For example, faculty can use GenAI tools such as ThingLink to support the development of unique and interactive simulations and case studies in diverse contexts designed to support and test students’ mastery of course competencies, including with attention to the nuances of operating in different contexts (i.e., rural or urban), cultures, and countries. AI tools can also be leveraged to support language translation and transcription of such course materials for a more authentic experience. In addition, faculty can also leverage AI tools such as ChatGPT to support development of negotiation or debate exercises on topics relevant to global health leadership, such as funding or policy negotiations between various stakeholders or countries. As more educational technology and AI tools proliferate the market, and as faculty begin or continue using such tools, the possibilities for advancement and innovation in global health education are vast and exciting and can lead to improved learner engagement and outcomes (43, 44). However, the integration of generative AI in health professional education must be accompanied by safeguards that protect academic integrity, ensure equitable access, and uphold ethical accountability in digital learning environments (45). Future research should examine how GenAI tools influence learning outcomes in global health, including consideration of any unintended effects, both positive or negative.

In conclusion, developing and implementing effective global health education is critical for improving both education and practice in global health. This study presents a unique global health education model which addresses many of the opportunities and challenges noted in the literature along with an evaluation of its effectiveness. Our intention in sharing this case study is to offer readers the opportunity to learn about and adapt this model in their own curricula and settings as desired as we collectively strive for improved global health education (we welcome readers to contact the authors if interested in learning more about the model or course materials). The more such models are implemented across various topics and various settings, the more we can achieve increased scalability and success in key components of global health training and practice.

## Data Availability

The raw data supporting the conclusions of this article will be made available by the authors, without undue reservation.
